# Psoas abscess in pregnancy: a review of the literature and suggestion of minimally invasive treatment options

**DOI:** 10.1007/s00404-023-06970-5

**Published:** 2023-02-25

**Authors:** Carolin Schröder, Elina Calite, Paul Böckenhoff, Thomas Büttner, Johannes Stein, Ulrich Gembruch, Brigitte Strizek

**Affiliations:** 1 Department of Obstetrics and Prenatal Medicine, University Hospital Bonn, Venusberg Campus 1, 53127 Bonn, Germany; 2 Prenatal Care Dr. Marquet and Bewermeyer, Muffeter Weg 37, 52074 Aachen, Germany; 3 Department of Urology and Paediatric Urology, University Hospital Bonn, Venusberg Campus 1, 53127 Bonn, Germany

**Keywords:** Psoas abscess, Double J-stent, Pregnancy, Urolithiasis, Hydronephrosis

## Abstract

**Aim:**

Less than a dozen cases of psoas abscesses in pregnancy have been described in the literature. We reviewed the literature when treating a patient with a psoas abscess after ipsilateral double J-ureteral stent placement (in the following: “double J-stent”) due to infected hydronephrosis.

**Methods:**

In January 2022, this review was searched using the Pubmed/MEDLINE database and the mesh terms “Psoas Abscess” AND “Pregnancy”. Studies were included in any language and of all years, describing a psoas abscess during pregnancy. When patients did not have a psoas abscess, the abscess occurred after pregnancy, or when there was no full text available, the article was excluded.

**Main results:**

Ten case reports about patients with psoas abscesses during pregnancy were included. The classical symptomatic triad of psoas muscle abscess included lower back pain, limping and persistent fever with daily spikes. However, in most cases, not all three symptoms can be found. Especially, fever is absent in more than half of the patients. Psoas abscesses are described between 13 and 39 weeks of gestation. Primary psoas abscesses with haematogenous spread are more common during pregnancy than secondary with spread per continuitatem. In the literature, the main reasons for psoas abscess are spinal tuberculosis, drug abuse or underlying diseases such as Crohn’s disease. It is not uncommon for the definite cause to be unclear. Regarding the patient's symptoms, pyelonephritis is often considered a possible aetiology. In general, the main treatment options include antibiotic treatment and abscess drainage. There is no higher caesarean section rate, and no negative outcome for the foetus has been described.

**Case presentation:**

In our patient, a 38-year-old obese Caucasian woman, who had received a left double J-stent for infected hydronephrosis at 15 weeks of gestation, we successfully treated a psoas abscess of 20 × 10 cm with a sonographically assisted abscess drainage and antibiotics. The further course of pregnancy and the elective repeat caesarean section at 38 + 0 weeks of gestation were without any problems. Double J-stent placement and laser stone lithotripsy during puerperium were performed because of recurrent urolithiasis.

**Conclusions:**

Although rare, psoas abscesses can occur during pregnancy, and it has often been treated surgically in the past. A psoas abscess as a complication after infected hydronephrosis and intervention during pregnancy has never been reported in the literature. Even for obese patients, minimally invasive therapy may be a treatment option that has rarely been reported in the literature.

## What does this study add to the clinical work

**Table:** 

This paper, describing the first case of a psoas abscess as a complication of infected hydronephrosis and double J-stent insertion in pregnancy, adds a new aetiology to the existing literature and shows minimally-invasive therapy as a feasible and safe treatment option during pregnancy.	

## Aim

At present, less than a dozen psoas abscesses are described during pregnancy. Psoas abscesses are divided into primary with haematogenous spread and secondary with spread per continuitatem. Pathogenic germs such as *Staphylococcus aureus* or *Mycobacterium tuberculosis*, but also underlying diseases such as chronic inflammatory bowel diseases or malignancy, are the leading causes. During pregnancy, the main reasons for psoas abscesses are spinal tuberculosis [1–3], drug abuse [4, 5] or pyelonephritis [6]. A psoas abscess in a patient with Crohn’s disease has been described only once [7]. In some cases, the exact cause remains unclear [2, 8, 9].

While treating a patient with a psoas abscess due to infected hydronephrosis after double J-stent insertion, we reviewed the literature. To the best of our knowledge, a psoas abscess in this context has never been reported and represents an unusual complication of a common diagnosis during pregnancy.

Psoas abscesses are challenging to diagnose due to heterogeneous symptoms. They should therefore be considered when a pregnant patient complains of persistent back or abdominal pain, weakness in the lower extremities, or fever of unknown origin.

## Methods

**Fig. 1:**
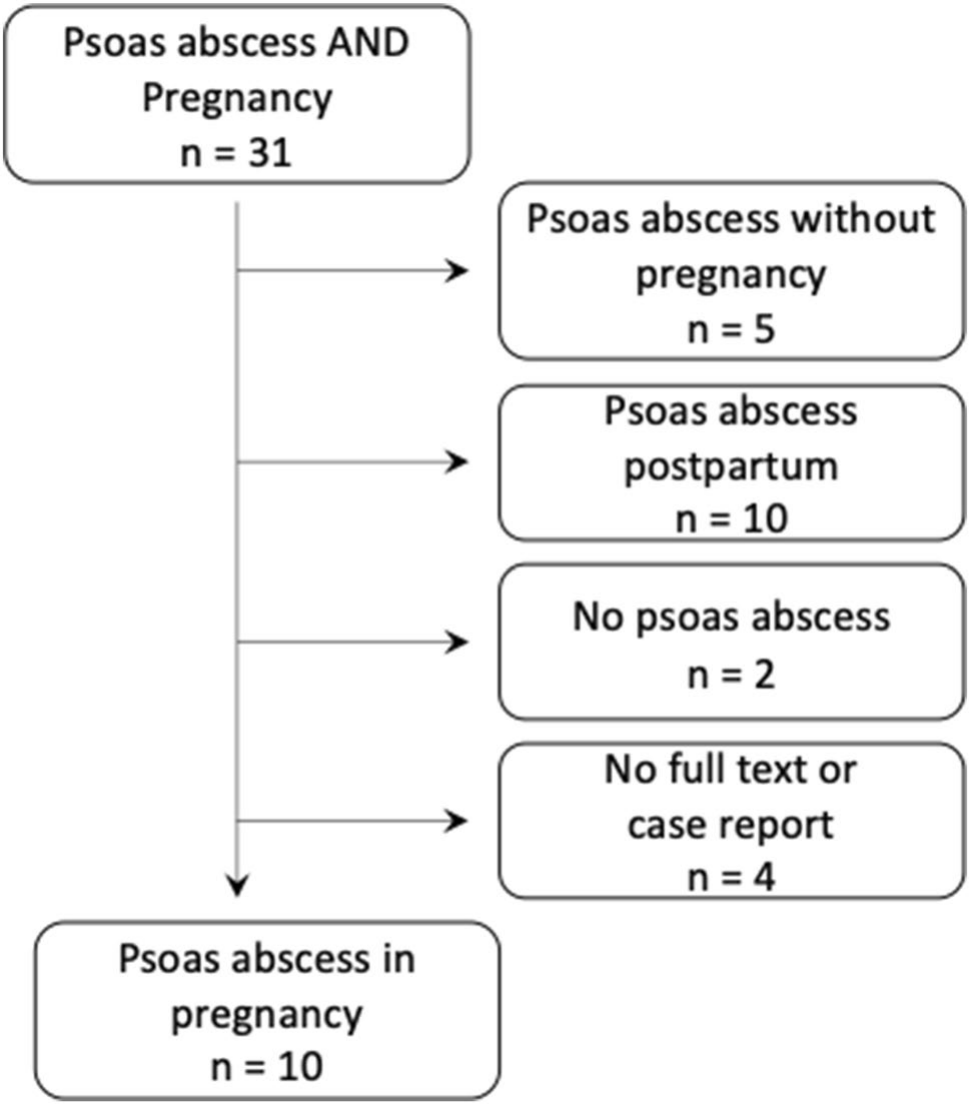
Research of literature

The search for this review was done in the Pubmed/MEDLINE database. Case reports were searched using the mesh terms "Psoas abscess" AND “Pregnancy”. Studies were not included when patients did not have a psoas abscess, when the abscess occurred after pregnancy, or when there was no full text available. Papers written in any language and of all years were included. The search was done in January 2022. In total, ten case reports fulfilled the criteria (Fig. 1).

## Case presentation

A left double J-stent was applied to an obese 38-year-old patient (BMI 45 kg/m^2^, G3/P1) for infected hydronephrosis at 15 + 3 weeks of gestation in another hospital. Because of severe discomfort, the double J-stent was removed at 23 + 1 weeks of gestation. The patient's symptoms improved after the removal but recurred soon afterwards, with newly emerging pain and weakness in the left leg, especially when raising the leg. Suspicion of an intervertebral disc affection led to magnetic resonance imaging (MRI). This revealed a 20 × 10 cm lesion adjacent to the left kidney, leading to the diagnosis of a psoas abscess. The patient was transferred to our clinic at 24 + 0 weeks of gestation. White blood cells (WBC) and c-reactive protein (CRP) were both elevated (17 × 10^9^/l and 278 mg/l). A calculated antibiotic treatment (piperacillin/tazobactam 1.5 g three times daily) was started, and ultrasound-guided percutaneous abscess drainage was performed. Fresh urinoma was ruled out by serum-identical creatinine in the punctate. MRI and ultrasound ruled out hydronephrosis at this time. Thus, further double J-stenting or percutaneous nephrostomy was not indicated. After accidental dislocation of the drain by the patent, a second abscess drainage had to be inserted into the residual abscess at 24 + 5 weeks of gestation. The drainage was flushed twice daily, and antibiotics were continued for 14 days. Microbiological examination revealed Prevotella bivia, sensitive to the antibiotic treatment already started.

**Fig. 2:**
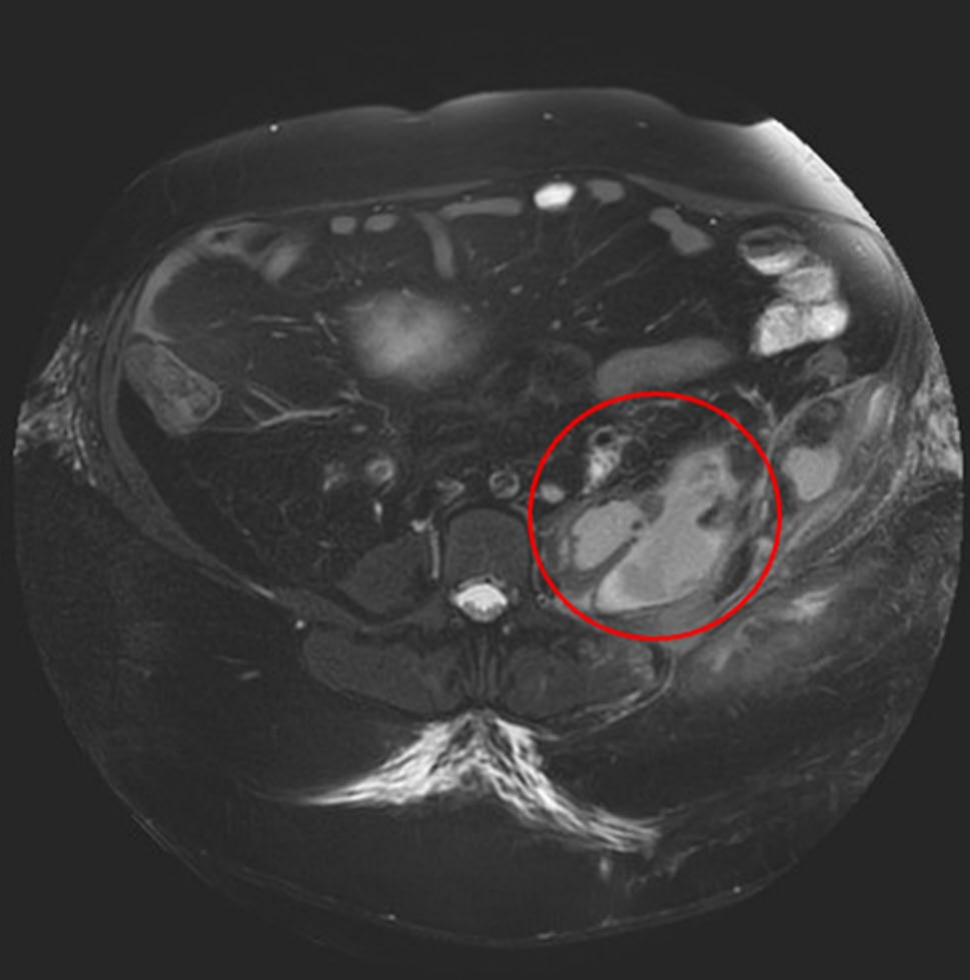
MRI in 25 + 4 weeks of gestation shows the regressed psoas abscess with a size of now 7 × 6 cm (previous: 20 × 10 cm)

**Fig. 3:**
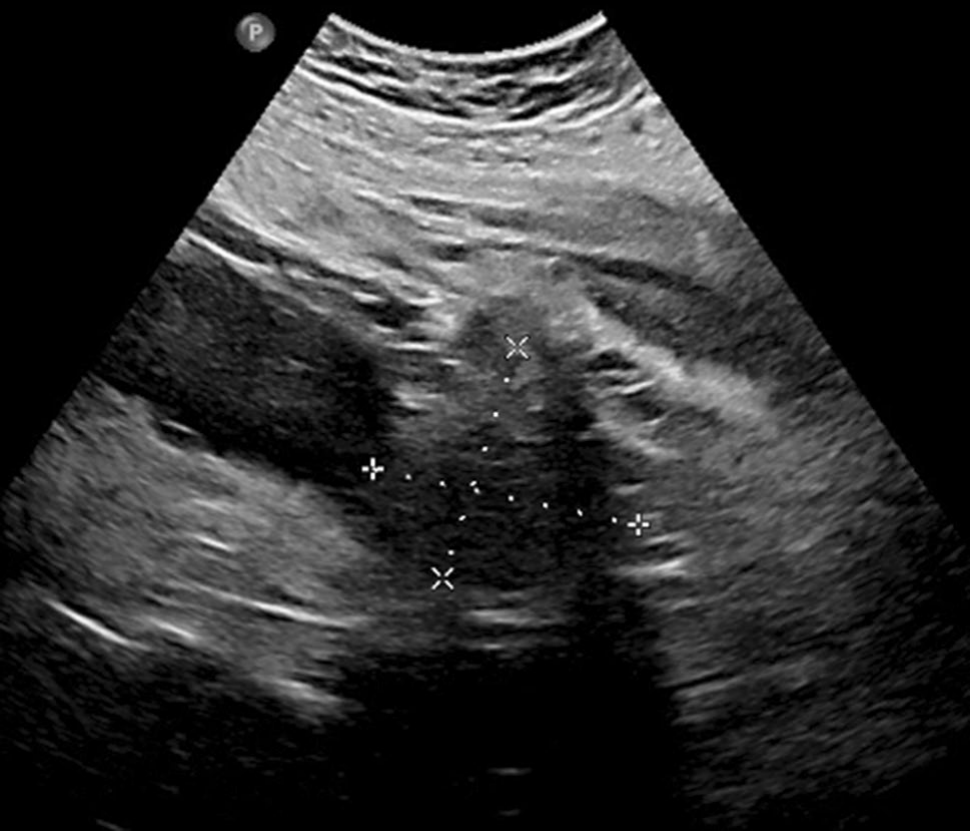
Sonography in 26 + 2 weeks of gestation: psoas abscess with 5 × 5 cm

Furthermore, the infection parameters were regressive in the following days. On Re-MRI at 25 + 4 weeks of gestation, the size of the psoas abscess had regressed compared to the external MRI, with a size of 7 × 6 cm (Fig. 2). There was no evidence of urolithiasis on the MRI. At 26 + 2 weeks of gestation, the abscess appeared sonographically to be approximately 5 × 5 cm in size (Fig. 3). The drainage was removed at 26 + 6 weeks of gestation with almost normalised infection parameters (WBC 10 × 10^9^/l, CRP 5 mg/l). The patient was free of fever at all times, with normal renal retention parameters, and could be discharged home.

The sonographic controls of the foetus at 32 + 0 and 36 + 0 weeks of gestation showed normal growth with good foetal development, normal amniotic fluid volume and Doppler indices. The patient was asymptomatic in the further course of the pregnancy.

Elective repeat caesarean section was performed at 38 + 0 weeks of gestation, with partial bilateral salpingectomy for tubal sterilisation after completed family planning. A female neonate, weighing 2850 g, was born with physiological pH of the umbilical cord artery and Apgar score. The postnatal course in our ward was uneventful.

During puerperium, computed tomography (CT) was performed due to colic-like flank pain. It showed evidence of bilateral urolithiasis, including a left ureteral calculus, leading to the re-insertion of a left double J-stent and subsequent ureterorenoscopic laser lithotripsy in another hospital. Examination of the stones revealed calcium oxalate stone composition.

## Results

**Fig. 4:**
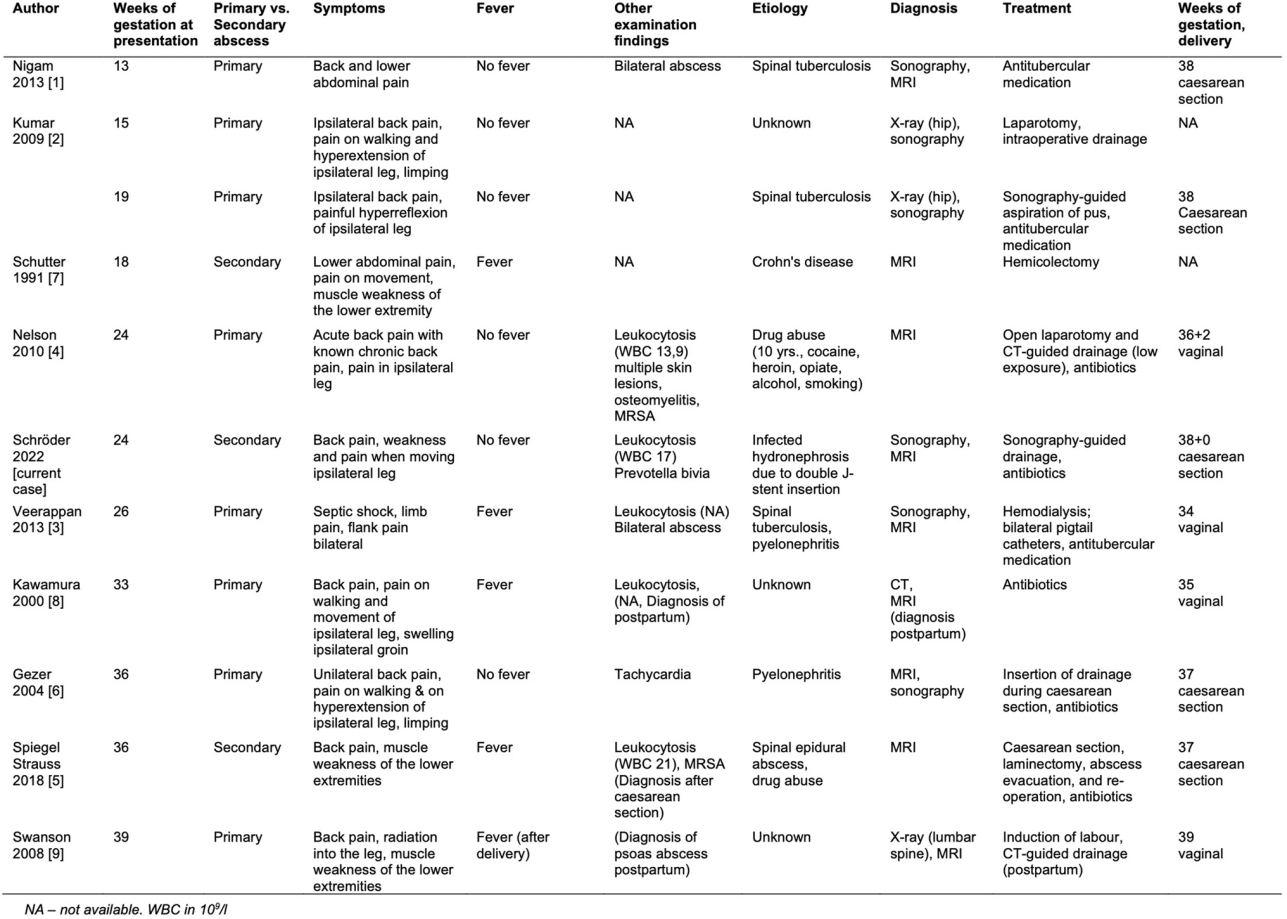
Review of literature

Ten case reports of patients with a psoas abscess during pregnancy were included (Fig. 4).

Both primary (*n* = 8) and secondary (*n* = 2) psoas abscesses have been described in pregnancy. Three case reports describe a primary psoas abscess in spinal tuberculosis, with bilateral psoas abscesses in two of three cases [1–3]. Furthermore, two cases of psoas abscesses associated with drug abuse have been described [4, 5]. There is only one case report of a psoas abscess in the setting of Crohn's disease in pregnancy [7]. It is not uncommon for the exact cause of the psoas abscess to remain unclear; pyelonephritis would often be considered a possible aetiology based on the symptoms and evidence of germs [2, 6, 8, 9].

Sonography and MRI are the methods of choice to confirm the diagnosis. X-ray examinations are rarely performed, however especially when the patient complained of pain during hip movement [2, 9].

Only four cases describe fever, back pain, and pain while moving the ipsilateral leg, defined as the triad of psoas abscess by Hermann Mynter in 1881 [3, 5, 7, 8]. Our case also shows the difficulty of diagnosis, especially when more common differential diagnoses are in the foreground. Our patient's complaints (pelvic pain at hip flexion, elevated inflammation parameters) were consistent with those of other case reports. However, the fever seems to be a missing symptom in more than half of the cases, especially in the patient's initial presentation.

Patients with psoas abscesses were treated with both antibiotics and surgical abscess drainage. Sonography or CT-guided drainage served as therapeutic options, especially in the early weeks of gestation, as well as ultrasound-guided aspiration of pus [2]. When the psoas abscess was evacuated during an operation, an intraoperative insertion of drainage was used [2]. In our case, we could place sonography-guided drainage in an obese patient, although it has not been described in the literature regarding the treatment of a psoas abscess so far. When using drainage, CT-guided procedures are mainly described [4, 9].

The literature shows no difference between the rate of caesarean sections compared to vaginal deliveries for patients with psoas abscess. In four cases, caesarean section was the mode of delivery [1, 2, 5, 6]. Surgical abscess evacuation and intraoperative insertion of drainage were also used during caesarean section [5, 6]. Vaginal deliveries are described in four patients with psoas abscesses [3, 4, 8, 9]. None of the cases described a negative outcome for the foetus. There was no delivery before 35 weeks of gestation (Fig. 4).

## Discussion

Symptomatic urinary retention and recurrent urinary tract infections in pregnancy are frequent, especially in late pregnancy.

Many patients have sonographic evidence of hydronephrosis at the end of their pregnancy (right > left) [10]. Both hormonal and mechanical factors cause this. In the case of kidney stones, these often resolve spontaneously, which is why conservative treatment such as analgesia and sufficient hydration should be the first-line treatment in the case of suspected or imaging-confirmed urolithiasis and symptomatic urinary retention. One problem during pregnancy is the non-availability of the standard urolithiasis diagnostic (low-dose native CT). The available options (MRI, ultrasound) lack sensitivity in detecting ureteral calculi. Measuring the kidney resistive index via pulsed-wave ultrasonography is a well-validated tool for evaluating obstructive ureteral disorders and may be utilised for this situation. If conservative therapy is not successful or there is an indication for immediate intervention, such as infected hydronephrosis, temporary or permanent urinary diversions should be discussed. The double J-stent is an option for temporary urinary diversion in hydronephrosis, but must be changed in 3–12 monthly intervals and therefore does not represent a permanent therapy.

Double J-stents are an effective and safe option for treating analgesia-refractory hydronephrosis in pregnancy, with rare complications. However, during pregnancy the intervention becomes substantially complicated in contrast to a non-pregnant condition: since the usual x-ray guidance and contrast enhancement imaging of the urinary tract by retrograde ureteropyelography cannot be used during placement, the less safe ultrasound-guided positioning must be performed. Hence, extravasations as in fornix rupture or ureter perforation remain invisible. Due to the possible post-interventional pain, urinary tract infections, discomfort and the risk of preterm labour, ureteral stents are not necessarily superior to conservative therapy with analgesia [10]. A percutaneous nephrostomy is an alternative to the double J-stent but is also associated with a significant impairment of quality of life due to external urine drainage and requires change at 4–6 weekly intervals. If there is no infectious situation, diagnostic and, if necessary, therapeutic ureterorenoscopy is possible if a ureteral stone is suspected during pregnancy [11].

In our patient, there was no evidence of a persistent ureteral stone at the time of treatment in our hospital due to the absence of indirect signs such as hydronephrosis. Consequently, further manipulation, such as repeat double J-stent insertion, percutaneous nephrostomy, or ureterorenoscopy, was not indicated.

Psoas abscesses can be divided into primary and secondary psoas abscesses: in primary abscesses, pathogenic germs spread along blood or lymph vessels, whereas in secondary psoas abscesses, the abscess spreads per continuitatem [2].

Hermann Mynter first described psoas abscesses in 1881 with the triad of back pain, fever, and conspicuous gait. Subsequent studies showed that not all three symptoms are present in all cases. *Staphylococcus aureus*, *Escherichia coli*, or *Mycobacterium tuberculosis* should be mentioned as the main pathogenic germs [12]. Primary psoas abscesses are more likely in younger patients (men > women). In contrast, older patients with underlying diseases such as chronic inflammatory bowel diseases are more likely to have secondary psoas abscesses [6]. To date, there have been no reports of psoas abscess following Double J-stent insertion. Psoas abscesses in pregnancy are very rare. There are some case reports of psoas abscesses after vaginal delivery, caesarean section and abortion curettage, but only a few cases of psoas abscesses during pregnancy (Fig. 4).

Psoas abscesses occur more frequently in the second and third trimesters but have been reported from 13 and 39 weeks of gestation. The high divergence of clinical symptoms can be explained by the particular location of the psoas muscle during pregnancy. Thus, back pain, pain during hip movement, an acute abdomen, fever and elevated CRP and WBC are described. However, they can be absent in some cases. It is not uncommon for the symptoms to be interpreted as pregnancy-related and only further clarified after delivery if they persist [9]. However, misinterpreting the patients' symptoms can lead to labour induction if the symptoms are interpreted as pregnancy-related. That is why there is a chance of missing a psoas abscess [9].

It is essential to mention that a psoas abscess is often diagnosed after delivery, especially in the later weeks of gestation [5, 8, 9]. Especially when symptoms persist postpartum, the initial diagnosis should be re-evaluated, and a more unlikely diagnosis should be considered. This also explains why patients are often misdiagnosed, and vertebral disc affection or pregnancy-related back pain is viewed more often [8].

Regarding the literature for tubo-ovarian or other abdominal abscesses, percutaneous drainages have been described as a safe and effective therapeutic option during pregnancy [13, 14].

Currently, no case report in the literature describes an association between a double J-stent and a consecutive psoas abscess in pregnancy. In general, several possibilities come into question as the cause of the psoas abscess in our case report.

From a urologic perspective, a fornix rupture or pyelonephritis are possible causes of the psoas abscess. Given the unavailability of retrograde ureteropyelography, fornix rupture or ureteral perforation could easily be missed during the externally performed double J-stent insertion. Although urinoma was excluded at the time of the patient's presentation in our hospital, the occurrence of a urinoma during double J-stent insertion, which later became superinfected, cannot be ruled out. To avoid this complication, we suggest changing to ureterorenoscopy or percutaneous nephrostomy in the event of unsuccessful guidewire insertion during sonographically guided DJ stent insertion.

It is recommended to consider a rarer differential diagnosis if symptoms persist after double J placement, especially if abnormal laboratory parameters are present. Sonography offers a good diagnostic option even in obese patients. If the findings are unclear, they can be supplemented by MRI. In the end, sonography and MRI examinations were able to determine the exact diagnosis and extent of the psoas abscess in our case.

The microbiological examination for pathogenic germs revealed Prevotella bivia; no other underlying diseases or intravenous drug abuse were present. Apart from obesity, there were no other risk factors, such as diabetes or immune disorder. It seems possible that endometriosis could also be another risk factor as it could increase the stiffness of tissue and hence increase the risk of perforation during double J-stent insertion. Our patient did not have an endometriosis and regarding the literature it is, however, not mentioned in other case reports if the patient had endometriosis or not. Despite the accidental dislocation of the drainage, the following course of the pregnancy was uneventful, and the delivery could be scheduled full-term. A new double J-stent insertion was not performed since, in the absence of hydronephrosis, there was no clear evidence of persistent urolithiasis, and the patient did not tolerate the previous double J-stent.

Since there was a re-appearance of symptoms with evidence of urolithiasis after birth, it can be assumed that this was also the cause of the initial complaints during pregnancy.

## Conclusions

As this case highlights, diagnosing and treating psoas abscesses during pregnancy can be challenging. It requires intensive interdisciplinary collaboration between prenatal physicians, urologists, and radiologists. Although hydronephrosis is common during pregnancy and is usually managed conservatively, it can cause severe complications in the infected stage. We report the first case of a psoas abscess as a possible but rare complication of infected hydronephrosis and double J-stent insertion in pregnancy, adding a new aetiology to the existing literature. In patients with persistent or worsening symptoms after double J-stent placement, an infectious complication should be considered even in the absence of fever. Although treatment was primarily surgical in the literature, ultrasound and/or MRI-guided drainage and antibiotic therapy should be considered as treatment options in pregnancy with potentially good maternal and foetal outcome.

## Data Availability

The dataset and materials used in this study are available from the corresponding author upon reasonable request.

## References

[1] Nigam A, Prakash A, Pathak P, Abbey P (2013). Bilateral psoas abscess during pregnancy presenting as an acute abdomen: atypical presentation. BMJ Case Rep.

[2] Kumar S, Malhotra N, Chanana C, Lal S (2009). Psoas abscess in obstetrics. Arch Gynecol Obstet.

[3] Veerappan I, Shanmugam A, Kumar S, Velayutham P (2013). Bilateral psoas and bilateral perinephric abscesses complicating acute pyelonephritis in pregnancy. Indian J Nephrol.

[4] Nelson DB, Manders DB, Shivvers SA (2010). Primary iliopsoas abscess and pregnancy. Obstet Gynecol.

[5] Spiegel Strauss TN, Pachtman SL, Rochelson B (2018). Bacterial spinal epidural and psoas abscess in pregnancy associated with intravenous drug use. Case Rep Obstet Gynecol.

[6] Gezer A, Erkan S, Erzik BS, Erel CT (2004). Primary psoas muscle abscess diagnosed and treated during pregnancy: case report and literature review. Infect Dis Obstet Gynecol.

[7] Schutter E, Schutter EM (1991). Psoas abscess in pregnancy: a case report. Geburtshilfe Frauenheilkd.

[8] Kawamura K, Sekiguchi K, Shibata S, Fukuda J, Tanaka T (2000). Primary psoas abscess during pregnancy. Acta Obstet Gynecol Scand.

[9] Swanson A, Lau KK, Kornman T, Wallace EM, Polyakov A (2008). Primary psoas muscle abscess in pregnancy. Aust N Z J Obstet Gynaecol.

[10] Çeçen K, Ülker K (2014). The comparison of double j stent insertion and conservative treatment alone in severe pure gestational hydronephrosis: a case controlled clinical study. Sci World J.

[11] Johnson EB, Pais VM (2012). Obstetric complications of ureteroscopy during pregnancy. J Urol.

[12] Shields D, Robinson P, Crowley TP (2012). Iliopsoas abscess—a review and update on the literature. Int J Surg.

[13] Kim YA, Chun K, Koh JW, Song HS, Kim H (2021). How to approach the rupture of tubo-ovarian abscess during pregnancy: a case report and literature review. J Obstet Gynaecol Res.

[14] Sherer DM, Schwartz BM, Abulafia O (1999). Management of pelvic abscess during pregnancy: a case and review of the literature. Obstet Gynecol Surv Oktober.

